# Nanoparticles in Chemical EOR: A Review on Flooding Tests

**DOI:** 10.3390/nano12234142

**Published:** 2022-11-23

**Authors:** Akram Al-Asadi, Eva Rodil, Ana Soto

**Affiliations:** 1 Cross-Disciplinary Research Center in Environmental Technologies (CRETUS), Department of Chemical Engineering, Universidade de Santiago de Compostela, E-15782 Santiago de Compostela, Spain; 2 Chemical and Petrochemical Techniques Engineering Department, Basra Engineering Technical College, Southern Technical University, Ministry of Higher Education and Scientific Research, Basra 61003, Iraq

**Keywords:** review, EOR, nano-fluid, core-flooding

## Abstract

The use of nanofluids is showing promise as an enhanced oil recovery (EOR) method. Several reviews have been published focusing on the main mechanisms involved in the process. This new study, unlike previous works, aims to collect information about the most promising nano-EOR methods according to their performance in core-flooding tests. As its main contribution, it presents useful information for researchers interested in experimental application of nano-EOR methods. Additional recoveries (after brine flooding) up to 15% of the original oil in place, or higher when combined with smart water or magnetic fields, have been found with formulations consisting of simple nanoparticles in water or brine. The functionalization of nanoparticles and their combination with surfactants and/or polymers take advantage of the synergy of different EOR methods and can lead to higher additional recoveries. The cost, difficulty of preparation, and stability of the formulations have to be considered in practical applications. Additional oil recoveries shown in the reviewed papers encourage the application of the method at larger scales, but experimental limitations could be offering misleading results. More rigorous and systematic works are required to draw reliable conclusions regarding the best type and size of nanoparticles according to the application (type of rock, permeability, formation brine, reservoir conditions, other chemicals in the formulation, etc.)

## 1. Introduction

The current global demand for fossil energy sources looks certain to continue for decades to come [1]. The need to move towards total independence from crude oil, switching to renewable sources of materials and energy, is undeniable. However, during the time needed to achieve the required technological development, crude oil is a practical necessity. To meet the worldwide needs, taking full advantage of production from current oil fields is therefore essential.

After primary and secondary recovery, the majority of the oil still remains inside the reservoir. Tertiary or enhanced oil recovery (EOR) methods look to recover this oil by the injection of gases, microorganisms, chemicals and/or thermal energy into the reservoir [2]. Chemical EOR methods are based on the injection of water combined with low concentrations of added chemicals. Commonly injected substances are surfactants (or alkaline/caustic chemicals that generate surfactants in situ) and polymers. Surfactants reduce the interfacial tension (IFT) between the oil and water. This reduction enhances the mobility of the oil retained in the pores, allowing it to be flushed out of the reservoir. Polymers increase the viscosity of water, thus reducing the difference between water and oil viscosities, leading to a more homogenous displacement. The chemicals can also be used to change the wettability of the rock. It has been shown that EOR methods are effective in recovering the difficult-to-access oil. However, the difference in viscosity between gas and oil in gas flooding, the unpredictable behaviour of microorganisms, the energy cost and risk of thermal methods, the cost of chemicals, the stability of formulations in the presence of salts, among many others, are problems that impede the optimal application of these methods.

At the beginning of this century, nanotechnology appeared as a new promising means to enhance oil recovery. There are many mechanisms involved when nanoparticles are used in formulations to extract oil [3,4,5,6,7,8,9,10,11,12,13,14]. Disjoining pressure is considered one of the key mechanisms of nano-EOR, where nanoparticles induce the detachment of oil from the rock surface while allowing the nanofluid to spread further [7]. Considerable improvement in oil recovery is usually attributed to wettability alteration effects caused by the nanoparticles, changing in the best case the wettability from strongly oil-wet to strongly water-wet. A synergistic effect in the reduction of IFT has also been shown in surfactant formulations with low concentrations of nanoparticles. Nanoparticles slightly increase the viscosity of the aqueous phase and, combined with polymers, enhance the rheological behaviour of the formulations. Furthermore, the capacity of nanoparticles to reduce oil viscosity and prevent asphaltene precipitation has also been shown. All this translates to successful viscosity control and an increase in the sweep efficiency. There are other advantages associated with nano-EOR. For example, nanomaterials can be used to increase the stability of surfactants and polymers in high-temperature and -salinity conditions. Adsorption of the injection formulation can be reduced by using the surface charge property of nanoparticles. Nanomaterials can also be used to plug some pores and thereby force the oil to exit from adjacent ones which were previously blocked. All these effects are obviously affected by nanoparticle type, size and concentration, and they strongly depend on the types of oil and rock, and reservoir conditions (salinity, temperature, heterogeneity). High nanoparticle concentration or diameter, for instance, can have a negative effect on reservoir permeability due to the blocking of porous media. The promising features of nanotechnology applied to EOR have led to a significant amount of research on this topic [3,4,5,6,7,8,9,10,11,12,13,14].

Several reviews [3,4,5,6,7,8,9,10,11,12,13,14,15] have been published collecting studies on EOR with nanoparticles. They shed some light on the main mechanisms involved and highlight the fact that many of them are not well-understood and that more work is required. However, as core-flooding tests are the most suitable techniques to ensure the efficacy of proposed formulations for EOR, those are likely the most interesting studies from the point of view of application. A recent review [16] investigated nanoparticle core flooding research from a data statistics perspective. Many statistical analyses are presented, and the main conclusion is that incremental oil recovery associated with the presence of nanoparticles usually is about 5% of the original oil in place (OOIP), in specific cases reaching as high as 30%. However, the paper does not focus on the details of the experimental studies, the main objective of this new work. Thus, this review offers information about core-flooding tests carried out with nanofluids: formulations used, experimental conditions, and enhanced oil recovery achieved. A critical analysis of the papers is considered out of the scope of this work. Firstly, an introduction to the interest of the topic will be offered. Secondly, the review will present the results of core-flooding tests using solely nanoparticles or functionalised nanoparticles; then, the combination with alcohols, surfactants, polymers or both chemicals will be considered. Finally, some conclusions focusing on practical application will be drawn.

## 2. Review

Initial proposals advocating the use of nanoparticles for EOR only involved their addition to the aqueous formulations used in secondary recovery. Due to the limited stability of the nanofluids found in most cases, the use of stabilizing agents such as alcohols was the natural following step. In an attempt to combine oil recovery methods, surfactants and polymers were used to design formulations with the advantages of these chemicals with nanoparticles. As an alternative, functionalisation enhances the stability and improves the properties of nanoparticles through surface modification, thus potentially offering a different route to obtaining practical results.

### 2.1. Nanoparticles

Since the first theoretical investigations [17] showed that suspensions of nanometre-sized particles affect the adhesion behaviour on solid surfaces with relevance to EOR, many studies have been published looking at the stability of nanofluids or wettability alteration [3,4,5,6,7,8,9,10,11,12,13,14,15]. However, it was not until the past decade that core-flooding tests were conducted to test the performance of nanofluids as injecting fluids for EOR, and to determine the optimum conditions for practical application. Lipophobic–hydrophilic silica nanoparticles (21–40 nm) were selected by Hendraningrat et al. [18] as an EOR agent for oil extraction from Berea sandstone reservoirs. Nanofluids with weight concentrations of 0.01, 0.05 and 0.1 wt% (pH decreasing with nanoparticle concentration) were used in core-flooding tests. Increases in nanoparticle concentration in brine (3 wt% NaCl) reduced water–oil IFT and contact angle in water-wet surfaces. In flooding experiments, after brine injection, EOR achieved ranged from 0 to 6.1% of the OOIP. High nanoparticle concentration caused blocking of the pore network, significantly in low-permeability rocks, which led to no additional oil recovery. Thus, nanoparticle concentration was found to be a critical parameter for nano-EOR. Fumed hydrophilic silica nanoparticles were also tested for the application [19]. In this case, the initial size of the nanoparticles was 7 nm (aggregation was observed by SEM), and the selected concentration was 0.05 wt%. Core-flooding experiments were carried out at different reservoir wettabilities and temperatures. Highest EOR (~6.6 %OOIP) was achieved at the highest temperature (80 °C) and with intermediate or oil-wet rocks. An extended post-flush with brine after nanoflooding led to incremental oil recoveries up to 4.9 %OOIP. These studies were later completed by the same authors, who analysed the influence of the chemistry of the injected water on oil recovery [20]. To investigate the effect of hardness, different water salinities and ionic compositions including divalent ions were tested. In general, the higher the salinity, the higher the improvement in wettability and oil recovery, this effect being more significant with the presence of divalent cations in the seawater. Despite these initial studies suggesting that nanoparticle concentrations higher than 0.05 wt% could lead to lower recoveries, Youssif et al. [21] increased SiO_2_ concentrations up to 0.5 wt%. Water flooding followed by silica nanofluid flooding was found to be more effective than using the nanofluid directly as secondary flooding. As expected, the incremental oil recovery did not mirror nanoparticle concentration, and the greatest oil recovery was achieved at 0.1 wt%. It was also found that permeability impairment increased as the injection rate increased, consequently making the optimisation of this parameter fundamental in EOR applications. The same nanoparticles, in this case around ~19 nm, were also tested for sandstone rocks by Lu et al. [22]. The brine used by these authors contained 7500 mg/L NaCl, and the permeability of the cores was very low. Similar conclusions to the previous studies were obtained. The improvement of the water-wet condition and the existence of an optimal nanoparticle concentration required to avoid the blocking of pore throats were confirmed. Due to the low permeability of the cores used, nanoparticle concentrations tested ranged between 5 and 30 ppm. The best results were obtained with a concentration of 10 ppm, at which a tertiary oil recovery of 10.3 %OOIP was achieved. It was shown that the viscosity and asphaltene content of the oil decreased as nanoparticle concentration increased. The effect of nanofluid imbibition on recovery was more significant at lower injection rates, leading to higher recovery. Moreover, the authors suggested a cyclic nanofluid injection to improve oil recovery. In contrast to previous studies, working with 0.5 and 1 wt% of 80 nm SiO_2_ nanoparticles, Nwufoh et al. [23] found that the highest concentration allowed for a better oil recovery; this is likely due to the flooding equipment used, a home-made sand-pack setup. However, the main development of this work was illustrating the possibility of using electrical resistance tomography as a non-destructive visualisation method to follow the dynamics of flow in sand-packs. The main recovery mechanisms were identified as IFT reduction and viscosity enhancement.

Recently, an attempt has been made to clarify the influence of nanoparticle concentration and salinity on oil recovery [24]. Studies were carried out with SiO_2_ (12 nm) concentrations of 0.02, 0.05, 0.07 and 0.1 wt% and different salinities. The conclusions were coincident with those obtained by Hendraningrat et al. [18,19], suggesting an optimum nanoparticle concentration of 0.05 wt%. Higher concentrations led to stability problems, with mechanical entrapment and pore plugging becoming more active mechanisms in oil recovery than those predominant at lower concentrations (wettability change and IFT reduction). Regarding the effect of salt concentration, the achieved EOR at 20,000, 30,000, and 40,000 ppm NaCl was 11, 8.3 and 11.2% OOIP with 0.05 wt% SiO_2_. Higher salinity increased stability problems, chemical EOR mechanisms were adversely affected, and at the same time, macroscopic mechanisms of pore throat plugging were enhanced. The authors concluded that an optimisation of all the parameters involved would be required to achieve maximum oil productivity. In another study, working at low flooding rates, Abhishek et al. [25] found no significant improvement in oil recovery from sandstones when silica nanoparticles were added to low salinity water. However, they demonstrated that nanoparticle adsorption on the mineral surface reduced mineral dissolution, ion exchange, loss of cementing mineral, and resistance to flow, thus reducing formation damage.

Aqueous formulations of silica nanoparticles were also proposed for EOR in carbonate reservoirs by Ahmadi and Shadizadeh [26]. Aerosil 200 (>99.8 wt% SiO_2_, 12 nm) concentration in the injecting fluids ranged from 500 to 10,000 ppm. Wettability studies were not performed, and the increment in oil recovery was justified due to mobility control caused by an increase in viscosity of the nanofluid. Core-flooding experiments were carried out at 100 °C. The nanofluid was injected as secondary flooding and achieved oil recoveries ranging from 57.2 to 65.2 %OOIP. An increase in nanoparticle concentration up to 0.6 wt% led to a remarkable additional recovery; however, a further increase to 1.0 wt% did not show a significant effect. Other flooding tests [27] carried out with hydrophobic SiO_2_ (Aerosil R 816) nanoparticles in carbonate reservoirs confirmed the improvement in oil recovery due to the presence of the nanomaterial. The concentration of SiO_2_ was varied from 0 to 10,000 ppm in water. The ultimate oil recovery was increased from 56 to 80.2 %OOIP, increasing the nanoparticle concentration up to 10,000 ppm. Nonetheless, as in the previous case [26], the increase in recovery at concentrations higher than 6000 ppm was very low. In another study [28], the impact of salinity on wettability alteration of carbonate rocks with hydrophilic mono-dispersed SiO_2_ nanoparticles was assessed. Contact angle measurement and spontaneous imbibition tests were used to show that both nanoparticle and electrolyte concentrations alter the wettability from oil-wet to water-wet, the phenomenon being more significant in the case of dolomite than in limestone substrate. Oil production in flooding tests was increased from 43.9 (before nanofluid injection) to about 55 %OOIP. However, several flooding stages were required. Using the same type of nanoparticles and rocks, the influence of different EOR scenarios on oil recovery, namely slug nanofluid injection, continuous nanofluid-flooding, and pre-soaking with nanofluids, was also assessed [29]. In this last scenario, oil recoveries of 78 and 71 %OOIP were achieved with nanoparticle concentrations of 0.1 and 0.3 wt%, values higher than those obtained with slug and continuous injections. The authors highlighted as advantages of the scenario: lower cost and lower permeability impairment, which reduced concerns about formation damage. As the main drawback, the unproductive period during the soaking must be highlighted.

The possibilities of TiO_2_ nanoparticles in EOR were analysed by Ehtesabi et al. [30]. The stability of several formulations containing 1 wt% TiO_2_ in the presence of different salts was tested, and precipitation problems were found with KCl and CaCl_2_. Flooding tests with sandstone cores were carried out with brine (5000 ppm NaCl), 0.01 wt% TiO_2_ anatase, 1% TiO_2_ anatase, and 1% TiO_2_ amorphous, and recovery factors were 49, 80, 42 and 23 %OOIP, respectively. At low nanoparticle concentrations, a homogeneous deposition of nanoparticles and nanorods (60 nm) onto the core plug surface that changed from oil-wet to water-wet was observed. Increasing the concentration, the presence of the nanorods also increased, resulting in pore plugging. To solve the problems of stability of TiO_2_ nanoparticles, Hu et al. [31] proposed the use of trisodium citrate dihydrate after testing the stability of the nanoparticles in the presence of NaCl with different stabilizers. Flooding tests with rutile-stabilized TiO_2_ nanoparticles in brine (0.1 mol/dm^3^ NaCl in deionized water) were carried out in water-wet Berea sandstone cores. While testing concentrations ranging from 5 to 500 ppm, the highest recovery at the breakthrough was achieved with 20 ppm (0.3 wt% stabilizer). However, 10 ppm led to the highest ultimate recovery factor, achieving an extraction of 41.8 in comparison to 30.3 %OOIP attained with water flooding. Mobility ratio change, rock wettability modification, and a log-jamming effect were the mechanisms proposed for the oil extraction.

Nanofluids consisting of 90 nm (Na_2_Ca_2_K_2_)_4_(H_2_O)_28_(Al_l8_Si_40_O_96_) nanoparticles synthesised from natural zeolites through high-energy ball milling followed by recrystallization were also tested for EOR [32]. Formulations were prepared in formation brine (Langgak Field in Indonesia) with low nanoparticle concentrations (1, 10 and 20 ppm), allowing the nanofluids to remain stable for a long period at low and high temperatures. The presence of the nanoparticles altered Bentheimer sandstone surface wettability from water-wet to stronger water-wet and decreased IFT between oil and injection fluid, the lowest IFT being achieved at 10 ppm. Core flooding tests at 60 °C led to additional oil recoveries of 6.9, 15.6, and 4.4 %OOIP for 1, 10, and 20 ppm nanofluids, respectively. Optimal performance, associated with the lowest IFT, was achieved at 10 ppm of nanoparticles.

It was shown that the adsorption of γ-Al_2_O_3_ nanoparticles (10–20 nm) on the calcite surface changes the wettability from oil-wet to water-wet [33]. Tested concentrations ranged from 0.1 to 1.5 wt%, with 0.5 wt% leading to the maximum change in contact angle. A core-flooding test at this concentration on carbonate rock increased oil recovery from 65 (water flooding) to 76.3 %OOIP with nanofluid tertiary flooding. The use of SnO_2_ nanoparticles was also proposed for this kind of rock [34]. As in the previous case, this nanomaterial was also able to change the wettability of the carbonate rocks from oil-wet to water-wet and to reduce brine-crude oil IFT. The comparison of the capability of extraction of synthetic seawater (containing divalent ions) with nanofluids containing 0.01, 0.05, 0.1, and 0.5 wt% SnO_2_ was carried out. The recovery factor was increased by 10, 13, 24, and 7%, respectively. For the highest concentration, rocks were found as water-wet, and pore plugging could have occurred.

A couple of works found in the literature [35,36] allow for the comparison of the performance of different nanoparticles for the same application. Bayat et al. [35] used aqueous solutions with 0.005 wt% of aluminium oxide (Al_2_O_3_, 40 nm), titanium dioxide (TiO_2_, 10–30 nm), and silicon dioxide (SiO_2_, 20 nm) for oil extraction from an intermediate-wet limestone sample at several temperatures. Al_2_O_3_ performed better than TiO_2_ and those nanoparticles better than SiO_2_, recovery increasing in all cases with higher temperatures. This was directly related to the adsorption, caused by the difference in surface charges of nanoparticles and limestone, with the highest adsorption in the case of silicon nanoparticles. Oil viscosity, IFT reduction, and wettability alteration were highlighted as the main mechanisms of recovery. The aforementioned nanoparticles together with zirconium dioxide (ZrO_2_), calcium carbonate (CaCO_3_), magnesium oxide (MgO), cerium oxide (CeO_2_), and carbon nanotubes (CNT) were tested in carbonate rocks [36]. A direct comparison of the performance of the different nanoparticles is difficult because different concentrations, stabilizers or surfactants were used with each type of nanoparticle. Nanofluids based on CaCO_3_ and SiO_2_ (with different kinds of chemicals) led to the highest recoveries of 8.7 and 7.7 %OOIP after water flooding. A structural disjoining pressure gradient was proposed as the mechanism responsible for wettability alteration.

Magnetorheological fluids are used to improve oil recovery because some properties such as stability in the presence of salts, apparent viscosity, yield stress, and storage modulus can be modified under magnetic fields. Esmaeilnezhad et al. [37] tested magnetite nanoparticles (<80 nm) in Berea sandstone core-flooding experiments. Permanent ring magnets were attached alongside the core sleeve to create a uniform magnetic strength throughout the core. A negative impact of salinity in the ferrofluid stability was observed, leading to precipitation problems even at very low nanoparticle concentrations. Thus, an injecting fluid consisting of 0.8 wt% magnetic nanoparticles in 5000 ppm NaCl was defined. Contact angle measurements showed the capacity of dispersion to change the rock wetting condition from water-wet to more strongly water-wet when using a light oil. However, no effect was observed when using a heavy crude oil. In line with this finding, flooding experiments in Berea sandstone with light oil showed an EOR (ferro-fluid and post-flush water flooding) 10.3% higher with the application of a magnetic field, whereas very limited enhancement was found in the case of the heavy oil. According to the authors, in the first case, the nanoparticles create a columnar structure in the direction of the applied magnetic field that operates like a piston and sweeps the oil from the pore channels. Even though the paper does not consider IFT reduction as a possible mechanism associated with nanofluid performance, Fe_3_O_4_ nanoparticles have shown a high capacity to reduce IFT under electromagnetic waves [38]. These and ZnO nanoparticles were able to reduce water–oil IFT, the former exhibiting greater performance in this regard. A simulation was carried out to determine if the presence of nanoparticles increased the sandstone response to the irradiation. Experimentally, a nanofluid consisting of 0.05 wt% Fe_2_O_3_ nanoparticles (<50 nm) in brine (11,000 ppm NaCl) was used to extract crude oil from sandstone rocks. After brine flooding, additional oil recoveries of 15.8 without and 29.4 %OOIP with irradiation were obtained, showing that the magnetic nanofluid coupled with electromagnetic waves can effectively enhance oil recovery.

Another method tested to increase the performance of nanoparticles alone for EOR is the use of smart water. This method is intended to alter rock/brine/oil interactions, such as wettability, through the modification of the ionic composition of water. Water flooding in carbonate reservoirs can be significantly improved by changing the salinity and ionic exchange of the injection brine. Thus, Mahmoudpour and Pourafshary [39] proposed the use of a hybrid method for EOR consisting of the use of smart water and nanoparticles. Different engineered waters, with the same ionic strength, were prepared by addition of magnesium, sulphate or calcium ions to a Persian Gulf brine sample. Then, 0.1 wt% of different nanoparticles (γ-Al_2_O_3_, TiO_2_, CaCO_3_, or SiO_2_) was dispersed in the different waters, but stable formulations were only found in the case of silica (80 nm). Different techniques (contact angle, zeta potential, attenuated total reflectance analysis, and environmental scanning electron microscope imaging) showed that the most successful alteration in the wettability of dolomite rock samples was achieved, adding sulphate ion concentration to the base brine. Sulphate anions neutralize the positive surface charge, facilitating the detachment of carboxylic groups by positive magnesium and calcium groups. The addition of nanoparticles to this smart water led to an additional oil recovery of ~11.3 %OOIP after the injection of formation water, a 4.5% higher oil recovery in comparison to the use of the engineered water alone as a tertiary EOR method. The main mechanisms associated with this hybrid method were: wettability alteration, reduction of nanoparticle adsorption, formation of microemulsions and IFT reduction, in addition to an increase in viscosity of the injected fluid. The introduction of smart water and silica nanoparticles has also been found useful to improve the recovery of asphaltenic oil from carbonate reservoirs [40]. In these reservoirs, due to the high percentage of asphaltenes, flow problems caused by the formation of stable emulsions are frequent. Smart water was prepared with 10,000 ppm of MgCl_2_, CaCl_2_, and Na_2_SO_4_ in deionized water. Nanofluids were prepared with 0.05 wt% SiO_2_ (25 nm). These nanofluids were efficient in preventing emulsion formation. Formulations capable of reducing IFT and improving wettability were tested in core-flooding experiments at 60 °C. First, formation brine was injected and then smart water including the nanofluid. In the first case, recovery increased from 12.9 to 36.6 %OOIP, in the second, from 19.5 to 65.1 %OOIP. Asphaltene deposition in porous media was observed.

Similar results to those mentioned above have also been presented at several conferences [41,42,43,44,45]. Details regarding oil, dispersion media (noting that many nanofluids were prepared in water instead of formation brine used to simulate reservoir conditions or secondary flooding), rock type, and temperature conditions for all the works considered in this section are shown in Table 1. The table includes publications with untreated, or modified but commercially available, nanoparticles.

### 2.2. Functionalised Nanoparticles

Surface engineering of nanoparticles can increase their stability, modify the rheological properties of the fluids, improve wettability conditions, and enhance IFT reduction. To improve nanoparticle stability, they are normally surface modified by anchoring different materials to enhance the electrostatic repulsive charges on their surfaces.

Silica is likely the most studied nanoparticle for nanoflooding and the most considered for functionalisation. The hydrophobic association of partially hydrolysed polyacrylamide complexed with silica nanoparticles has been proposed to improve the performance of water soluble polymers in high-temperature and high-salinity oil reservoirs [46]. These nano-hybrids were used in flooding experiments (silica sand) at 85 °C and were compared with the use of polymers alone. Nano-hybrids were prepared with 0.5 wt% of polymer and 0.5 wt% silica in synthetic brine (containing divalent cations). Tertiary oil recoveries of 5.4 and 10.6 %OOIP were found with the polymer and the nano-hybrid, respectively. Besides the improvement of the long-term stability of the polymer, the interactions among nanoparticles and the polymer reinforce the structure of the latter and increase its viscosity and elasticity.

The use of silicate-based nanoparticles was also proposed by Sagala et al. [47]. They synthesised, using a soft hydrothermal method and triethoxy(octyl)silane, different forms of nanopyroxenes (silicate-based nanoparticles): un-functionalised or neutral pyroxene, half hydroxyl functionalised or Janus pyroxene, and fully hydroxyl functionalised hydrophobic pyroxene. Formulations containing the different nanoparticles at a fixed concentration of 0.005 wt% were tested for EOR. Fully functionalised nanoparticles led to the best results in IFT reduction and wettability alteration; thus, they were tested for EOR from sandstone rock. An additional recovery of 10.6 %OOIP was achieved after brine flooding. Similarly, functionalised nanoparticles were synthesised by Cao et al. [48] by reacting 3-aminopropyltriethoxysilane and octyltriethoxysilane with nano-silica particles. Their hydrophobic group content was adjusted with the amount of octyltriethoxysilane. Amphiphilic nanoparticles were able not only to reduce IFT and improve wettability, but also to generate a notable emulsification effect. The highest hydrophobicity of the nanoparticles led to the highest recovery in core-flooding tests. After water flooding, achieved EOR with nano-silica (1000 mg/L) was 2.6 in comparison with 10.3 %OOIP obtained with the best nanomaterial.

A silica nanocomposite (SiO_2_@Montmorilant@Xanthan) was synthesised from natural products and tested by Nazarahari et al. [49] in both sandstone and carbonate cores. Formulations with different concentrations (100, 250, 500, 1000, 1500, and 2000 ppm) were prepared in water, 250 ppm producing the highest reduction of IFT. This concentration was also selected as optimal to change the wettability of carbonate rocks, whereas 1000 ppm was used to change the wettability of the sandstone rocks. The first formulation led to an EOR (after secondary recovery with seawater) of 11.7 and the second to 15.8 %OOIP, for the respective types of rocks. Also from natural products (pomegranate seed), a ZnO/SiO_2_/xanthan nanocomposite was prepared and tested for EOR in carbonate reservoirs [50]. The best results in IFT reduction were achieved with a suspension consisting of 2000 ppm of nanocomposite in dilute seawater. Injection of the low-salinity polymeric fluid after water flooding, in a carbonate core, led to an additional 19.3 %OOIP recovery.

The functionalisation of Fe_3_O_4_ was also tested for practical application [51,52]. Fe_3_O_4_@Chitosan nanocomposites were prepared by Rezvani et al. [51]. A reduction in IFT between seawater and crude oil was found, improving with increasing temperature and nanocomposite concentration. Wettability was also improved. Moreover, a significant reduction in oil viscosity was achieved when adding the nanomaterial. A formulation consisting of 0.03 wt% Fe_3_O_4_@Chitosan in seawater showed an increase of 10.8% of the oil recovery factor, compared to seawater, in carbonate sand. In another work [52], Fe_3_O_4_ nanoparticles were functionalised co-precipitating acidic aqueous solutions of FeCl_2_ and FeCl_3_ mixtures containing citric acid monohydrate in alkaline medium. An anionic polymer was required to achieve good nanomaterial stability at high salinity and temperature. Suspensions of the anionic polymer–citrate-coated Fe_3_O_4_ nanoparticles were able to reduce water–oil IFT and contact angle. After three-step flooding (water–nanofluid–water) at 85 °C in sand-pack porous media, a formulation consisting of 400 ppm of functionalised nanoparticles in brine (a mixture of natural formation water and seawater) allowed for a recovery factor enhancement of the secondary flooding of 19.7 %OOIP. The same nanoparticle concentration was tested in a carbonate core. Again, in this case, the displacement efficiency during nanofluid injection was very low, and a third water flooding stage was required to achieve an additional oil recovery of about 28 %OOIP. However, a significant increase in differential pressure, arising from formation damage due to nanoparticle aggregation on pores, was observed.

Amphiphilic molybdenum disulphide (KH550-MoS_2_) nanosheets were synthesized, using a hydrothermal approach, and they were tested for EOR [53]. Formulations with a very low concentration of the nanomaterial (50 mg/L) were able to decrease IFT, change contact angle and significantly enhance emulsion stability. Core flooding tests were carried out for dynamic adsorption and oil recovery experiments at Changqing oilfield reservoir conditions (55 °C and salinity ~7.8 × 10^4^ mg/L). Tertiary oil recoveries with MoS_2_ and KH550-MoS_2_ nanofluids in low permeability oilfield outcrop cores were 3.5 and 14 %OOIP, respectively. The depressurization rate for the latter was larger. According to the authors, this is likely due to adsorption on the pores forming a smooth nanofilm during transport, reducing flow resistance of the displacement fluid.

Graphene-based amphiphilic Janus nanosheets drastically reduced IFT in a saline environment (4 wt% NaCl and 1 wt% CaCl_2_). Formulations with very low nanoparticle concentrations (0.005 and 0.01 wt%) were tested in sandstone cores and enhanced oil recovery factors ranged between 6.7 and 15.2 %OOIP [54]. The authors indicated that the generation of elastic interfacial films may be the reason for the high oil recovery.

Combining the use of smart water and functionalised nanoparticles can also improve EOR. The use of a synthetic nanocomposite (amine/organosiloxane@Al_2_O_3_/SiO_2_) and smart water was proposed by Habibi et al. [55] for wettability alteration of carbonate rocks. By increasing the concentration of active ions (Ca^2+^ and SO_4_^2−^) in synthetic seawater, the contact angle decreased, and the rock wettability shifted towards water-wet. Adding nanoparticles, the contact angle dropped sharply, but a maximum concentration of 50 ppm was able to provide stable nanofluids. Core flooding tests were carried out by consecutive injection of seawater, smart water (double concentrations of Ca^2+^ or SO_4_^2−^), and smart water with nanoparticles (50 ppm). The increase in sulphate concentration led to higher recoveries than in the case of calcium-enriched engineered water. Oil recoveries obtained with this nanofluid and sulphate-enriched water in the different injection stages were 45.0, 55.0 and 62.5 (smart water + nanofluid) %OOIP. With the use of smart water, oil recovery improved compared to seawater, and the combination with the nanocomposite showed the best performance.

Details regarding oil, dispersion media, rock type, and temperature conditions for all the works considered in this section are shown in Table 2.

### 2.3. Alcohol + Nanoparticles

Alcohols can improve the miscibility of oil and water as well as the solubility of hydrophobic nanoparticles. Consequently, the combination of these solvents with nanomaterials has also been proposed as an EOR method. The role that different fluids (water, brine, ethanol and diesel) play as nanoparticle dispersing agents in EOR formulations was analysed by Ogolo et al. [56] by sand flooding under surface conditions. Interesting conclusions were obtained by these authors, with a definitive role of the dispersing agent on the positive or negative effect of the nanoparticles in oil recovery. The best performances were obtained with Al_2_O_3_ when formulations were based on water or brine, and with silane-treated SiO_2_ or hydrophobic SiO_2_ when ethanol was used as solvent. In the first case, nanoparticles were responsible for a significant reduction of the oil viscosity; in the second case, the change of wettability produced by the nanomaterial and a reduction in IFT generated by the alcohol were the main EOR mechanisms.

Regarding core-flooding experiments, formulations consisting of a partially hydrophobic fumed silica (Aerosil R 816) in ethanol (3 g/L) were tested in core plugs of sandstone rocks [57]. After water flooding, additional oil recoveries of 25.4 and 14.6 %OOIP were achieved with light and intermediate oils, respectively. Nanoparticles helped to reduce water–oil IFT and produced a change in wettability towards oil-wet more efficiently in the case of the light oil. Ethanol was also selected as dispersion media in the case of polysilicon nanoparticles [58,59]. Onyekonwu et al. [58] tested hydrophobic and neutrally wet nanoparticles dispersed in ethanol and hydrophilic nanoparticles dispersed in water. Contact angles and core-flooding tests were carried out. It was found that formulations with ethanol not only changed rock wettability but also produced a significant reduction in IFT. Those formulations (with 3 g/L or lower nanoparticle concentration) were proposed for water wet formations (30,000 ppm of salinity) and light- or intermediate-grade oils. EOR efficiencies ranged from 29 to 39 %OOIP (total recoveries from 55 to 80%) depending on the type of oil and polysilicon. On the other hand, formulations with untreated polysilicon nanoparticles in water did not achieve a significant recovery. A similar study was carried out by Roustaei et al. [59], achieving additional oil recoveries of 32.2 and 28.6 %OOIP with hydrophobic and neutrally wet nanoparticles, respectively. No formation damage was detected according to pressure drop data.

Propanol has also been proposed as a nanoparticle-dispersing agent. Formulations of Fe_2_O_3_, Al_2_O_3_ and SiO_2_ treated with silane nanoparticles (1.5 g/L) in this alcohol were tested by Joonaki and Ghanaatian [60] through core-flooding tests with sandstone rocks. The nanoparticle concentration was optimised via contact angle measurements. All the nanoparticles were able to decrease the IFT between water and oil, and they changed the rock wettability from water-wet to neutral-wet. Additional oil recoveries after brine flooding were 20.2, 17.3 and 22.5 %OOIP with Al_2_O_3_, Fe_2_O_3_ and SiO_2_, respectively. The injection of nanofluid after propanol flooding or from the beginning improved these results, but it will also increase the cost of recovery.

Details of the core-flooding tests presented in this section are shown in Table 3.

### 2.4. Surfactant + Nanoparticles

Chemical flooding with surfactants is well known to have huge potential in EOR processes. These chemicals are used to achieve significant reductions in the IFT between oil and water. This reduction enhances the mobility of the oil retained in the pores, allowing it to be flushed out of the reservoir. The combination of nanoparticles with surfactants has shown excellent performance in oil recovery, since the nanomaterial can reduce adsorption of surface-active agents on the rock surface.

The combination most widely studied in this category involves sodium dodecyl sulphate (SDS) as a surfactant and SiO_2_ nanoparticles [61,62,63,64,65]. This is the combination suggested by Eshraghi et al. [61] who proposed the use of a formulation optimised according to IFT reduction (2150 ppm SDS and 0.1 %wt SiO_2_ in dilute reservoir brine). The formulation not only improved the results of the surfactant in IFT reduction but also altered the wettability of sandstone rocks to strongly water-wet. Achieved oil recoveries with water flooding, surfactant flooding, and the optimised formulation (secondary mode) were 53%, 73%, and 80 %OOIP, respectively. According to these results, the improvement in performance justifies the addition of nanoparticles to the surfactant. The combination of the same surfactant with hydrophilic silica nanoparticles (Aerosil 200) was proposed by Bagrezaie and Pourafsharey [62]. In this case, the formulation was prepared in water, and the nanoparticle concentration was optimised to obtain minimal surfactant adsorption. Thus, a formulation containing 2500 ppm of surfactant and 1000 ppm of nanoparticles in water was selected for core-flooding tests. Similar conclusions to the previously mentioned study were obtained, oil recoveries in this case being 71.4, 82.0, and 94.7 %OOIP with water, surfactant and nanofluid flooding, respectively. More details about rocks and the experiments are given in Table 4. A further study on this system [63] was carried out focusing on surfactant adsorption in the same kind of rock (sandstone). Increasing silica concentration up to 0.3 wt%, surfactant adsorption was significantly reduced. However, the reduction of the surfactant adsorption was limited when the silica nanoparticle concentration increased to 0.4 wt% due to agglomeration of the nanoparticles. A higher nanoparticle concentration than in the previous studies was proposed (0.2 wt% SDS and 0.2 wt% silica) to carry out flooding tests. The use of nanofluid followed by a water post-flux led to an EOR of about 9.1 %OOIP, improving the 4.4 %OOIP achieved using the surfactant alone.

A formulation consisting of synthetic brine with divalent ions (3% NaCl), Aerosil 300 (hydrophilic amorphous silica) and SDS was also proposed by Zallaghi et al. [64] for EOR. Stability of nanoparticle suspensions was analysed and found to decrease exponentially with the increase in salinity and nanoparticle concentration, the problem worsening at higher temperatures. Thus, the use of SDS was required to improve the stability of the nanofluids in harsh conditions. The SiO_2_ nanoparticles not only altered wettability from oil-wet to water-wet conditions but also reduced the IFT. Sandstone core plugs were used for flooding experiments. Additional oil recoveries achieved with nanofluids without surfactant ranged from 2.4 to 11.7 %OOIP. As shown in many of the works presented in this and previous sections, excessive nanoparticle concentration led to blocking of pore throats, reducing oil recovery. These authors [64] suggested the use of 2000 ppm for rock permeabilities close to 150 mD. A higher EOR (14.5 %OOIP) was found with a nanofluid consisting of 0.2 %wt SiO_2_ + 0.04 %wt SDS, these results improving with the injection of the formulation as secondary recovery. Another study [65] focused on a comparison of the performance of hydrophilic (Aerosil 300) and hydrophobic (Aerosil R 816) silica nanoparticles when combined with this sulphate surfactant. As in the previous cases, surfactant adsorption was significantly reduced by the presence of the nanoparticles, and an adequate concentration of the nanomaterial also allowed for a noteworthy reduction in the IFT between water and oil. Flooding experiments were performed in unconsolidated sandstone packs. The best results were obtained with hydrophobic nanoparticles, due to a lower adsorption and a higher viscosifying capacity. The injection of an SDS solution (2000 ppm) allowed for a recovery of about 45% of OOIP that subsequently increased by 20.4% after the injection of the nanoparticle-augmented surfactant solution (2000 ppm SDS, 10,000 ppm R 816) and water post-flux.

Other nanofluids based on the common SDS anionic surfactant have also been proposed for EOR [66,67]. Cheraghian and Nezhad [66] suggested the use of nanofluids with nano-clay (Iranian sodium bentonite) to enhance recovery of heavy oil from sandstone rock. Nano-clay performed better than clay in terms of reduction of surfactant adsorption. Oil recovery by surfactant flooding with SDS (1800 ppm) was 46.2%. The combination with 2.5 wt% clay only showed an increase of about 0.1%. However, when the surfactant was used with 2.5 wt% nano-clay, an increase of about 6.1% was achieved. Another work proposed the combination of the same surfactant with ZnO nanoparticles [67] to improve the extraction of an organic fluid (*n*-dodecane) from a porous medium (sandstone). A formulation consisting of 0.2 wt% SDS and 0.05 wt% ZnO in water was selected as the most stable among the different mixtures tested. Oil recovery percentage increased in the tertiary phase from 16% (only SDS) to 35% (SDS + ZnO). A significant increase in differential pressure was detected due to retention of ZnO nanoparticles in the porous media. The authors highlighted the role of the surfactant as a stabilizing agent, and according to simulation studies, the dominant mechanism was the improved displacement efficiency.

Three different surfactants, dodecyltrimethylammonium bromide (DTAB), SDS, and Triton X-100 (TX-100), were combined with graphene oxide and modified graphene oxide (Janus graphene oxide, JGO150 and JGO400) nanosheet materials, to achieve ultra-low IFT [68]. The most promising combination (1000 mg/L DTAB, 100 mg/L JGO400 in formation brine) was used as an injection fluid in flooding tests with artificial cores, achieving an additional oil recovery of 19.5 %OOIP. This value clearly improved the results obtained with nanoparticles or surfactant alone.

Protection of the environment is currently a worldwide priority; thus, it is not surprising that the use of surfactants obtained from natural products is the focus of many recent studies [69,70,71]. Leaves and fruits of the Cedr or Zizyphus Spina-Christi (Middle East tree) contain many saponin compounds that are natural surfactants. Both Cedr extract (CE) and nanosilica were shown to help the reduction of IFT of kerosene and aqueous solutions [69]. Further, 1000 ppm of nano silica and 5 wt% CE in 100000 ppm brine (NaCl) were selected as the injection fluid in core-flooding tests. Adding nano-silica caused an increase in oil recovery from 53 (by secondary flooding with CE solution) to 74 %OOIP (nanofluid) in sandstone core. Using another natural surfactant (anionic rhamnolipid), Khademolhosseini et al. [70] demonstrated that nanoparticle morphology can also have a significant impact on EOR. They proposed the use of the above-mentioned natural surfactant in combination with silica nanoparticles for practical application. Micromodel tests (defined through a central composite design method) were carried out to analyse the effect of the shape of the nanoparticles, in addition to other variables, on oil recovery. The best performance was found with spherical nanoparticles since a higher uniformity resulted in better distribution and more effective interactions with crude oil components. Core flooding tests with an optimised formulation consisting of 110.8 ppm biosurfactant, 97.5 ppm spherical nanoparticles and 1.17 wt% NaCl led, in carbonate cores, to an additional 5.1 %OOIP after brine flooding (3%NaCl). The main mechanisms involved in oil recovery improvement were wettability alteration to water-wet, IFT reduction, and mobility ratio improvement. Later, the use of rhamnolipid with silica was proposed by Wang et al. [71] who also tested other natural surfactants such as sophorolipid and surfactin. Stable systems were achieved working with biosurfactant and nanoparticle concentrations below the critical micelle concentration and 1000 mg/L, respectively, in 3 %wt NaCl solutions. Various biosurfactants and compositions of nanofluids were tested in flooding experiments with low permeability cores of Berea sandstone. Several injection schemes were also tested. Additional oil recoveries up to 25% were achieved. The best results were found with rhamnolipid, an optimised nanoparticle concentration depending on the permeability of the core, and a cyclic mode of injection.

Surfactant-augmented functional silica nanoparticles were proposed for EOR by Zhou et al. [72]. To that aim, silica nanoparticles were modified with amino groups on the surface and then electrostatically linked to the Soloterra 964 surfactant molecules on the surface to form a nanocomposite. The nanofluids remained stable for more than a month in harsh conditions (65 °C and 15 wt% NaCl). A formulation consisting of 0.05 wt% SiO_2_-NH_2_-nanoparticles, 0.2 wt% Soloterra 964 surfactant and 15 wt% NaCl was able to change the wettability of Berea rocks from oil-wet towards water-wet. Core flooding tests with Berea core samples led to additional recoveries of 6.9, 11.1, and 22.4 %OOIP using nanoparticles, surfactant and nanofluid formulations, respectively, followed by brine flooding. The combination of amine-terminated silica nanoparticles with other surfactants was also tested. The nonionic surfactant laurel anolamide was selected by Liu et al. [73] to form thermodynamically unstable but kinetically stable Pickering emulsions. Flooding tests in three-layered (of different permeabilities) heterogeneous rectangular cores were carried out. After water flooding, the nanofluid led to an EOR of 26.4 %OOIP in a core of 100/200/500 mD, and 29.2 %OOIP in a core of 50/100/300 mD. Several mechanisms are responsible for this improved oil recovery: solubilisation and mobility control of the Pickering emulsions, IFT reduction and wettability alteration of the rock surface.

Despite the great number of laboratory studies involving nanoparticles for EOR, their application in real reservoirs is very limited. The first trial application was carried out in a Colombian oil field (December 2019–April 2020) with a formulation designed by Franco et al. [74]. The authors published their results with undefined commercial anionic surfactants (SA and SB) and nanoparticles (CNA and CNB). It was shown that surfactant adsorption on the rock could be reduced by at least 40% due to nanoparticle addition. Crude oil recovery tests were conducted under reservoir conditions and using synthetic cores with the reservoir mineralogy. A nanofluid formulation containing 1000 and 100 mg/L of the SA surfactant and CNA nanoparticles was tested in core-flooding experiments, achieving a better performance in displacement tests compared to the use of surfactant alone, with a tertiary recovery increase of almost 18%. The cumulative incremental oil field production was nearly 30,035 bbls for two injection patterns by 19 May 2020.

One of the main difficulties of carbonate reservoirs lies in their heterogeneity. Pore throat size distribution drastically affects reservoir fluid saturation. For that reason, Rezaei et al. [75] studied the relationship between pore throat size and the oil–water relative permeabilities of reservoir rocks. IFT and contact angle measurements were carried out to design an optimised formulation comprising 0.01 wt% of SiO_2_ and 0.01 wt.% of alpha olefin sulfonate (AOS) surfactant in brine (180 g/L NaCl). According to the authors, the homogeneity of the pore sizes is more important than the dimensions of the pores in conventional water flooding. The use of the optimised formulation in core flooding tests led to enhanced recoveries (after brine flooding) of 2.5 and 8.6 %OOIP for carbonate core plugs with homogeneous and heterogeneous pore throat sizes, respectively.

The synergistic effect between nanoparticles and surfactant molecules in EOR for both sandstone and carbonate reservoirs was shown by Kuang et al. [76]. The authors worked with several nanoparticles: SiO_x_, Al_2_O_3_, and TiO_2_, and surfactants: oleic acid, polyacrylic acid, a non-ionic surfactant (linear alcohol, C_9−11_, ethoxylate), an anionic surfactant (ammonium alkyl, C_6−10_, ether sulphate), a cationic surfactant (n-alkyl dimethyl benzyl ammonium chloride), and SDS. Some associations were found not to be beneficial but rather quite the opposite. SiO_x_ nanoparticles led to the best stability, the behaviour of Al_2_O_3_ and TiO_2_ nanofluids being dependent on the concentration of the chemicals. All the tested nanofluids were able to reduce IFT; however, in some cases (cationic surfactant and oleic acid), the wettability of the rock changed towards less water-wet, or even oil-wet. The arrangement of nanoparticles/nanoaggregates and surfactants was responsible for the different changes in wettability. Non-ionic nanofluids were found to be the most effective for sandstone and carbonate reservoirs, the reduction in IFT being the main mechanism involved. From imbibition tests, a formulation containing SiO_x_ (0.1 wt%) and the non-ionic surfactant (0.1 wt%) in brine (1mM NaCl) was selected to carry out core-flooding experiments at high pressure and temperature. Recovery enhancements (in comparison to brine flooding) up to 6.2% were achieved in Berea sandstone, while in carbonate samples, the recovery was unsuccessful due to the presence of calcium ions.

The influence of silica nanoparticles on the behaviour of two amino-acid surfactants (lauroyl-cysteine and lauroyl-arginine) for prompting oil recovery, via reduction of IFT and alteration of wettability, was analysed by Asl et al. [77]. Nano-surfactant solutions with 2000 ppm L-Arg/4500 ppm L-Cys and 1000 ppm SiO_2_ were used to extract oil from carbonate cores. In core-flooding tests, brine solution (10,000 ppm NaCl) was injected simulating secondary recovery, and 39.1 and 44.2 %OOIP were obtained. Additional oil recoveries of 13.1 and 12.7 %OOIP were achieved with L-Arg and L-Cys formulations, respectively. Nanosurfactant flooding was compared with the use of surfactant or nanoparticles alone and presented the highest performance.

A combination of EOR methods was proposed by Rezaei et al. [78] who integrated surfactant, alkali and nano-fluid flooding. Alkalis (Na_2_CO_3_, Na_2_B_4_O_7_) and nanoparticles (SiO_2_, ZnO and nano-clay cloisite) were combined with four anionic surfactants: SDBS, linear alkylbenzene sulfonic acid, coconut di-ethanol amide, and cocamido propyl betaine (CAPD). All the additives, as well as NaCl salt (in concentrations lower than 5 wt%), helped to further reduce the IFT and contact angle of the oil droplet on dolomite rock samples in comparison with the pure surfactants. Flooding experiments, on carbonate sister core samples, with a formulation consisting of CAPD (250 ppm), Na_2_CO_3_ (1000 ppm) and NaCl (198,000 ppm), led to an additional oil recovery of 19.7 %OOIP, in comparison to 12.2 %OOIP achieved with a formulation containing CAPD (250 ppm), SiO_2_ (1000 ppm) and NaCl (198,000 ppm). In a following paper, the effects of initial wettability and different flooding scenarios on oil recovery from carbonate rocks were also analysed by these authors [79]. A formulation consisting of 0.03 wt% linear alkylbenzene sulfonic acid and 0.1 wt% SiO_2_ nanoparticles in brine (20 wt% NaCl) was used to that aim. As in the previous case, both surfactant and nanoparticles facilitated the process of wettability alteration and IFT reduction. Moreover, it was shown that the presence of the nanomaterial reduced the adsorption of the surfactant on carbonate rock surfaces. Several flooding tests were carried out, and in this case, the water-wet core plugs showed lower oil recovery than the oil-wet plugs. The authors attribute this to a higher tendency of oil droplets to become isolated in larger pores of the water-wet porous media. The best flooding scenario was the secondary (nanofluid followed by brine flooding), achieving total oil recoveries higher than 60 %OOIP.

Instead of aqueous surfactant solutions, the injection of microemulsions or emulsions is sometimes proposed as an EOR method. Pickering emulsions are usually proposed to plug the high-permeability water channels in rock reservoirs and improve EOR; however, their long-term stability is still a challenge. Winsor III micro-emulsions (desired for EOR applications because they are associated with ultra-low IFT values) were found with a methyl ester sulfonate surfactant derived from Jatropha oil and lysine-grafted silica nanoparticles [80]. A significant EOR (33.6 %OOIP) was achieved in a sand-pack (manually prepared) with a microemulsion system consisting of 8000 ppm jatropha surfactant +1% nanoparticle + 2% NaCl. A Pickering emulsion with great stability under harsh reservoir conditions was achieved by means of a surfactant widely used for many applications, SDBS, and anisotropic aluminium oxide hydroxide slice-shaped (AlOOH) nanoparticles [81]. At a fixed 1 wt%, SDBS concentrations lower than 5 mM led to oil-in-water emulsions. The successive increase in the surfactant concentration led to water-in-oil, which then switched back to oil-in-water emulsions again. An oil-in-water emulsion (water:dodecane ratio=1:1vol) containing 3mM SDBS and 1 wt% AlOOH showed high tolerance in a wide range of shear rates, temperatures and electrolyte concentrations, and was selected for core-flooding experiments. The Pickering emulsion, followed by extended water flooding, led to an additional 33 %OOIP after initial water flooding. Another oil-in-water emulsion, in this case composed of the surfactant hexadecyltrimethylammonium bromide (CTAB) and SiO_2_ nanoparticles, was proposed by Pei et al. [82] for the application. The authors found that the addition of the nanoparticles not only increased emulsion stability but also significantly increased its bulk viscosity. Higher nanoparticle concentration led to smaller emulsion droplets. The prepared emulsions were shear-thinning across the entire shear rate range tested, and nanoparticle concentration was found as a useful tool to adjust the viscosity. A formulation consisting of water:biodiesel ratio = 9:1wt, 0.4 wt% SiO_2_ + 0.1 wt% CTAB was compared with a formulation only containing 0.1 wt% CTAB in flooding tests on unconsolidated man-made cores. Tertiary oil recoveries (nanofluid + extended water) in the first case ranged from 17 to 50 %OOIP, much greater than in the second case where maximum recoveries of just 23 %OOIP were achieved. Further studies were carried out by these authors [83] with the same formulation in order to optimise the injection conditions. Incremental oil recovery increased, injecting greater emulsion volumes, with economic reasons being definitive in selecting the suitable volume. Low rate and continuous injection were found more effective than high rate and cyclic injection in order to raise additional oil recovery. Qin et al. [84] synthesised silicon oxide nanoparticles in situ by a sol-gel reaction in a microemulsion consisting of: triton (surfactant), *n*-dodecyl *β-*D-maltoside (surfactant), *d*-limonene (oil) and 2-propanol (co-surfactant) with a weight ratio of 2:2:1:0.8. Brine (1 M CaCl_2_) was added up to a content of 99.5%. Flooding tests were carried out in Arkose (heterogeneous sandstone). After water flooding, additional oil recoveries achieved were 20.0 and 34.3 %OOIP with the microemulsion and nanoparticle-stabilised microemulsion, respectively. The best results of the latter were associated with the emulsification of oil into small droplets, where nanoparticles and surfactants synergistically interacted at the interface.

The application of magnetic/dielectric nanoparticles activated by electromagnetic waves for EOR is a technique that has recently been gaining attention [85]. Dielectric nanoparticles, such as ZnO, can be polarised under electromagnetic irradiation, which can reduce the mobility ratio, aggregation and water-oil IFT, and alter wettability, among other EOR mechanisms. Adil et al. [86] proposed the combination of ZnO nanoparticles with the surfactant SDBS for EOR. An injection fluid consisting of 0.1 wt.% nanoparticles and 0.025 wt% surfactant in brine (3 wt% NaCl) was tested in unconsolidated sand-pack core-flooding experiments. After secondary flooding, electromagnetic-assisted nanoflooding (1PV nanofluid + extended water flooding) with 55.7 and 117.1 nm nanoparticles led to additional oil recoveries of 14.0 and 16.1 %OOIP, in comparison to 13.8 and 15.3 %OOIP without the waves. According to the authors, the method could be promising for high-temperature reservoirs.

Details of the core-flooding tests presented in this section are shown in Table 4.

### 2.5. Polymer + Nanoparticles

The combination of nanoparticles and polymers generates a series of advantages for EOR. The polymer increases the viscosity of the aqueous phase, thus reducing the difference between water and oil viscosities, leading to a more homogenous displacement. Nanoparticles can enhance the rheological properties of the polymer whilst inhibiting its degradation and facilitating oil detachment through disjoining pressure.

Partially hydrolysed polyacrylamide (HPAM) is a water-soluble polymer used extensively in EOR despite its poor heat tolerance and lack of salt resistance. Improvement of these parameters has been tried by combination with different kinds of nanoparticles. Polymeric nanofluids with Al_2_O_3_ nanoparticles were proposed by Gbadamosi et al. [87] and were compared to SiO_2_-HPAM systems. The rheological properties of the polymer solution improved due to the presence of nanoparticles, while degradation of HPAM was inhibited. For a 2000 ppm HPAM solution (25 mol. % degree of hydrolysis), it was found that 3.41 wt% NaCl was the critical salinity threshold above which viscosity reduction was insignificant, and 0.1 wt% nanoparticle concentration was found to be the optimal choice due to the maximum achieved in apparent viscosity (all tests were carried out at 27 °C). Wettability experiments were also conducted to confirm an alteration of the porous media towards water-wet. Water flooding, at 90 °C, yielded a 30.2 %OOIP recovery in a sandstone core. Tertiary flooding with HPAM was 26.3 %OOIP while values with SiO_2_-HPAM and Al_2_O_3_-HPAM (0.2 wt% polymer and 0.1 wt% nanoparticle) were 33 and 37.6 %OOIP, respectively. An increase in pressure drop was noted with the use of nanoparticles. The same conclusions were obtained in a second publication by these authors regarding these systems [88].

HPAM and three sulfonated polyacrylamides with different sulfonation degrees were combined with hydrophilic silica, generating several suspensions that were rheologically characterised and applied to EOR by Elhai et al. [89]. It was shown that the bridging flocculation of the suspensions increased with nanoparticle size and concentration, but also with electrolyte charge and concentration. However, it decreased with pH and polymer concentration. Water-wet sandstone-based core plugs (from southern Iranian reservoirs) were used in recovery studies. The best results were achieved with brine (containing NaCl and CaCl_2_) flooding (52.6 %OOIP), followed by the injection of a formulation with 2000 ppm HPAM and SiO_2_ nanoparticles (0.1 wt%), leading to 8.0 %OOIP of additional recovery.

The combination of HPAM with TiO_2_ nanoparticles was also proposed [90]. A formulation consisting of 3150 ppm HPAM and 2.3 wt% TiO_2_ was prepared in synthetic brine. A medium-permeability reservoir sandstone core was used in the flooding tests. After water flooding (9.5 %OOIP), nanofluid and extended water flooding led to an additional oil recovery of 43.3 %OOIP. The use of higher or lower nanoparticle concentration resulted in worse extraction performance. This is due to a higher apparent viscosity of the formulation with the optimised nanoparticle concentration. Similar tests were carried out by these authors using sodium bentonite (<50nm) with HPAM [91]. As in the previous study, 3150 ppm of HPAM was the optimised concentration according to rheological and oil recovery studies. In this case, 0.9 wt% was the threshold value for nanoclay concentration. Nanoclay polymer flooding enhanced oil recovery by a factor of 5% in comparison to polymer flooding.

The design of polymers resistant not only to temperature and salinity but also to extreme pH conditions was carried out by Haruna et al. [92]. To that aim, different acrylamide co/terpolymers were produced via free-radical polymerization. Formulations were prepared adding multi-wall carbon nanotubes (MWCNTs) to American Petroleum Institute (API) brine or solutions with different pH values (acidic pH = 3 or alkali pH = 11). The polymer was added last. Formulations containing 1000 ppm polymer and 1000 ppm nanotubes were tested in flooding experiments at 85 °C with Ottawa sand-pack. The polyampholytic terpolymer and polyelectrolyte copolymer containing negative sulfonate groups showed the best performance in alkali and API brine, respectively, with EOR values of 10.8 and 14.8 %OOIP. The authors point to the viscosity as the main factor responsible for high oil recovery. The same research group, aiming to solve the problems of stability of SiO_2_/PAM nanocomposites in harsh conditions, proposed the modification of SiO_2_ nanoparticles with (3-aminopropyl)triethoxysilane to increase dispersion stability [93]. Polymer and nanocomposites were prepared in situ via free-radical polymerisation. The proposed nanocomposite, besides being more stable, also led to an increase in oil recovery. API brine was used for secondary recovery, from Ottawa sand-pack, and additional oil recoveries of 7.7, 12.7 and 17.5 %OOIP were obtained with 0.1 wt% PAM, SiO_2_/PAM, and modified-SiO_2_/PAM injections, respectively, followed by chase water. Similar results were obtained with formation brine from the Ghawar field in Saudi Arabia.

Hendraningrat and Torsaeter [94] proposed the use of hydrophilic metal oxide nanoparticles (TiO_2_ and Al_2_O_3_) in tertiary oil recovery for sandstone reservoirs. The use of a polymer, polyvinylpyrrolidone, was required to avoid the agglomeration of the nanoparticles. The addition of this chemical increased the stability of the formulations even at high temperature. Tests were also carried out with nano-suspensions of SiO_2_ for comparative purposes. Among the three nanoparticles tested, TiO_2_ led to the best results. After brine flooding, 56.67 %OOIP was recovered. EOR with an aqueous formulation containing 0.05 wt% of these nanoparticles and 1 wt% polyvinylpyrrolidone in brine (30,000 ppm NaCl) led to 11.11% of additional oil recovery. A final brine flooding led to a total recovery of 76.67 %OOIP. Contact angle measurements confirmed that titanium produced a higher wettability alteration to water-wet than the other nanoparticles.

The effect of alumina nanoparticles on polymer flooding using biopolymers was investigated by Orodu et al. [95]. Two different biopolymers were selected: potato peel starch (PSP) and gum Arabic (GA). The stability and rheology of formulations containing PSP at different salt concentrations and temperatures were analysed. They exhibited strong shear thinning behaviour. In the case of GA, the study focused on the effect of temperature. Several core-flooding tests were carried out to test the performance of the nanocomposites in EOR. In the case of the PSP formulations, additional oil recoveries (depending on the nanoparticle concentration) ranging from 10.2 to 12.4 %OOIP were achieved after a tertiary flooding using the polymer. A classical EOR scheme was selected for GA formulations, and additional oil recoveries ranging from 5.6 to 7.8 %OOIP were achieved after secondary flooding with brine. As in the case of previous studies, the role of nanoparticles as stability enhancers and viscosity modifiers was highlighted.

Rheological behaviour, wettability alteration, IFT reduction, and emulsification mechanisms were studied for suspensions of silica nanoparticles and xanthan gum in synthetic brine [96]. Polymer concentrations ranged from 1000 to 5000 ppm and SiO_2_ from 0.1 to 0.5 wt%. The presence of nanoparticles improved all the tested properties, resulting in a very effective nanofluid. The greatest oil recovery, from Berea sandstone, was achieved at the highest polymer concentration and 0.3 wt% SiO_2_. Additional oil recoveries were 20.8 %OOIP at 30 °C and 18.4 %OOIP at 80 °C. A similar study was carried out with the same nanoparticles and guar gum [97]. Flooding a sandstone core with a formulation containing 0.2 wt% SiO_2_ and 4000 ppm guar gum (with a post-water flooding) allowed for an additional oil recovery of 44.3 %OOIP.

Nanofluids have also been tested in fractured systems [98]. To that aim, a quartz tube with a copper sheet placed in the centre was filled with borosilicate glass beads and sintered in a furnace. In this case, polyethylene glycol 8000 was selected as the polymer, due to its relative stability with respect to electrolytes and temperature, and SiO_2_ was used as the nanomaterial. The nanofluid composition was 0.277 wt% in brine (0.25 wt% NaCl). Oil displacement in the water-wet bead-pack was 66.6 %OOIP in secondary and 23.8 %OOIP in tertiary flooding. However, in the case of oil-wet bead-pack, a low additional oil recovery was found (6 %OOIP). The authors concluded that nanofluid injection may be very effective for EOR in fractured media in water-wet to mildly water-wet systems.

The number of studies on nano-polymer flooding in carbonate rocks is currently very limited [99,100]. Rezaei et al. [99] proposed the combination of HPAM with surface-modified clay nanoparticles. These were functionalised by the inclusion of specific tetrahedral redundants between polymer chains. The addition of these nanoparticles to the polymer improved its resistance to salinity and temperature. A maximum value of viscosity, independently of temperature, was found with a nanoparticle concentration of 0.1 wt%; thus, this was the composition selected for the formulation with 2000 ppm of polymer. Flooding tests were carried out with unconsolidated carbonate sand-packs. With an oil recovery of ~33 %OOIP, the injection of the nanofluid increased the oil recovery achieved using just the polymer. Ali et al. [100] took advantage of the synergy of EOR mechanisms, using TiO_2_/SiO_2_/poly(acrylamide) nanocomposites and smart water for the application. The nanoparticles were synthesised in an eco-friendly manner using pomegranate seed extract and were later mixed with PAM. A smart-nanofluid consisting of 1500 ppm of nanocomposite and 5000 ppm of CaSO_4_, and CaCl_2_ salts demonstrated the highest performance in increasing oil recovery from 36.0 after water flooding to 46.5 %OOIP.

Details regarding oil, dispersion media, rock type, and temperature conditions for all the works considered in this section are shown in Table 5.

### 2.6. Surfactant + Polymer + Nanoparticles

All the chemicals able to help enhanced oil recovery (surfactant, polymer and nanoparticles) were combined by Sharma et al. [101]. The addition of SiO_2_ nanoparticles (0.5, 1.0, 1.5, and 2.0 wt%) to aqueous solutions containing 1000 ppm HPAM (30–35% degree of hydrolysis) and SDS (0.14 wt%) resulted in several benefits for EOR: increase in viscosity formulation, reduction of IFT, and change in the wettability towards water-wet. Moreover, it was found that oil recoveries with polymer alone, or polymer plus surfactant, decreased with increasing temperature, the nanofluids thus showing more stable behaviour with this variable. Additional oil recoveries via injection of the nanofluid and a brine post-flux ranged from 19.3 to 24.7 %OOIP (increasing with nanoparticle concentration and depending on temperature). These results clearly improved the performance of the use of just nanoparticles, polymer or polymer plus surfactant. Nanoparticle retention in the sand-pack, with a decrease in permeability, was also shown.

Corredor et al. [102] proposed nanoparticle inclusion to address the limitations of polyacrylamides in harsh reservoir conditions. Aqueous formulations containing polyacrylamide-grafted SiO_2_, TiO_2_, and Al_2_O_3_ nanoparticles (0.2 and 0.4 wt%), SDS (0.1 wt%), HPAM (0.4 wt%) and NaCl (1.0, 2.0, and 3.0 wt%) were tested for EOR in sandstone rocks at 25 °C. A low performance was achieved with SiO_2_ and Al_2_O_3_ nanoparticles due to their adsorption on the sand grains. However, the addition of 0.4 wt% titanium-grafted nanoparticles to HPAM solutions was able to increase the cumulative oil recovery of the polymer solution between 5 and 7%, independently of the NaCl concentration. The combination of nanoparticles, polymer and surfactant was also considered by Son and Lee [103]. In this case, three colloidal silica (diameters: 7, 12, and 22 nm) and fumed silica (diameter: 12 nm) nanoparticles were used to analyse the influence of particle size on IFT and oil recovery from sandstone rocks. Formulations containing 0.5 wt% nanoparticles, 0.5 wt% anionic polymer (PSS-co-MA) and 0.1 wt% surfactant (3-(N,N-dimethylmyristylammonio)propanesulfonate) in brine were tested for the application. It was found that smaller nanoparticles enhanced viscous behaviour, more effectively decreased IFT, and more strongly adsorbed to the rock, changing the oil contact angle. However, fumed nanoparticles led to lower oil recovery due to aggregation problems. The best recoveries were achieved with 7 nm colloidal nanoparticles, obtaining 78.2 %OOIP when injecting the formulation at 25 °C.

Harsh conditions in reservoirs is a typical problem addressed by Zhou et al. [104] using a nano-composite formed of polymer nanoparticles and a betaine-type zwitterionic surfactant. The nanoparticles were synthesised by means of a nanoprecipitation method with poly[(9,9-dioctylfluorenyl-2,7-diyl)-alt-co-(1,4-benzo-{2,1′,3}-thiadiazole)] and poly(styrene-co-maleic anhydride) cumene terminated. The developed nanofluid significantly reduced water/oil IFT and changed the wettability of an oil-saturated Berea core from oil-wet to water-wet. In core-flooding tests, after brine flooding (46 %OOIP), surfactant and nanofluid injection (both followed by brine flooding) led to additional oil recoveries of 11.4 and 20.0 %OOIP, respectively.

The combination of nanoparticles with surfactants and polymers frequently leads to the proposal of the use of oil-in-water Pickering nanoemulsions as injection fluids for EOR [105,106,107,108]. In this research line, nanoemulsions containing oil, SDS mixed with a conventional detergent (0.22 wt%), nanoparticles (clay or SiO_2_, 1 wt%) and polyacrylamide (1000 ppm) were compared with the use of polymer alone and surfactant for EOR by Sharma et al. [105]. To that aim, several core flooding experiments were performed in Berea sandstone rocks at high pressure and temperature. The incorporation of nanoparticles led to a higher reduction of IFT and limited the decrease in viscosity with temperature; thus, the use of Pickering nanoemulsions improved oil recoveries compared to the traditional surfactant–polymer method. A relatively larger retention of SiO_2_ than clay (smaller size) nanoparticles was detected. Formulations containing 0.22 wt% SDS, 1.0 wt% SiO_2_ or clay and 1.0 wt% polyacrylamide (followed by chase water) led to additional oil recoveries ranging from 17 to 21 %OOIP. Another Pickering nanoemulsion, formulated using light mineral oil, SDBS, carboxy methyl cellulose (CMC), and SiO_2_ nanoparticles was characterised through rheology measurements and used to extract oil from sand-pack cores [106]. The composition of the different chemicals was optimised via apparent viscosity and IFT measurements. The selected nanoemulsion (oil volume fraction = 0.1, 825 ppm SDBS, 1.5 wt% CMC, 0.25 wt% nanoparticles) led to an additional oil recovery of 24.8 %OOIP (including water post-flux). A pseudoplastic nanoemulsion composed of heptane, a gemini surfactant (N,N′-bis((dimethyltetradecyl)-1,6-hexanediammonium bromide)), HPAM, and nanoparticles, was proposed by Pal et al. [107] for the application. Surface tension was used as a criterion to define the optimal formulation. There was a minimum in this property at 0.10% surfactant (beyond this concentration, surface tension was observed to increase due to the non-availability of vacant sites). Polymer and nanoparticle addition increase and decrease, respectively, the property. The lowest surface tension was obtained for nanoemulsion systems comprising 0.10% surfactant, 0.05% HPAM and 0.025% SiO_2_. The addition of nanoparticles improved the stability of the nanoemulsions and increased their apparent viscosity. Moreover, the rock (sandstone) wettability was changed from intermediate-wet to water-wet. The use of this formulation, followed by chase water, in core-flooding experiments, allowed for an oil recovery of 26.3 %OOIP after conventional water-flooding. A further study on the mechanism of flow of the same nanoemulsion, and subsequent displacement of trapped crude oil through interconnected pore throats and unswept regions was carried out by these authors [108].

Recently, ionic liquids (salts with melting or transition temperatures below 100 °C) have been gaining a lot of attention for many applications. Surface-active ionic liquids (SAILs) are being extensively applied for EOR, their main advantages being their tunability and stability in harsh environmental conditions [109]. Only two papers have been recently published where the synergism of using SAILs and nanoparticles was exploited [110,111]. Stability and IFT studies were carried out to design a formulation consisting of 0.05 wt% Al_2_O_3_, 1.0 wt% PVP, 0.5 wt% 1-dodecylpyridinium chloride in brine 0.5 wt% NaCl. The formulation achieved an EOR of 12 %OOIP in carbonate rocks, thus improving the results obtained using just the SAIL or the SAIL plus polymer [110]. The same methodology was used to design a stable formulation at higher salt content (5 wt% NaCl) [111]. With the SAIL 1-dodecyl-3-methylimidazolium chloride (0.05 wt%) and the same nanoparticle and polymer concentrations, an EOR of 14.8 %OOIP was achieved. Both formulations were able to reduce water-oil IFT and improve wettability conditions.

Details of the core-flooding tests presented in this section are shown in Table 6.

## 3. Conclusions

In this work, a compilation of papers involving core-flooding tests with nanoparticles has been offered aiming to show the state of the art of the extraction capability of nano-EOR. Combinations of nanoparticles with chemicals (surfactants and/or polymers) have also been considered. This review allows for the establishment of some conclusions.

- The literature on the topic is sometimes confusing. Many tests (stability, rheology, IFT, contact-angle, etc.) are carried out, sometimes in the absence of salts and other times in their presence, before the final flooding experiments. As a consequence, the exact composition of the formulation tested in these studies is frequently unclear, especially regarding the presence of salts in the nanofluids. Future works should clearly define the formulation used in oil recovery studies.
- The greatest challenge for EOR in general, and for nano-EOR in particular, is the design of a stable formulation at harsh conditions (temperature and salinity) also considering the presence of divalent ions. This is a difficult task common to all EOR methods, but it is likely more difficult in the presence of nanoparticles. Very few works address the whole problem.
- The simplest and most cost-effective nano-EOR method involves the use of nanofluids consisting only of water or brine and common nanoparticles. Additional oil recoveries achieved with these systems are comparable to those achieved with other EOR methods and justify, in principle, their use to promote oil recovery.
- Even though the number of papers published with simple nanofluids is rather significant, there is not enough information to select the best type and size of nanoparticles according to the application (type of rock, permeability, formation brine, reservoir conditions, etc.). Critically, the number of studies in carbonate rocks is very limited.
- Size and more importantly concentration are key factors that seem to be more critical than the type of nanoparticle for success in practice. The nanofluid concentration must be optimised according to core permeability. Excessive concentration generates aggregation and blocking problems, thus limiting oil extraction and creating pressure problems. Nanoparticle concentration higher than 0.2 wt% is rarely recommended.
- SiO_2_ nanoparticles are cost-effective and are consequently the most often proposed for the application. Colloidal are preferred to fumed nanoparticles in order to avoid aggregation problems. Al_2_O_3_ is sometimes proposed for harsh environments.
- Many experimental studies have confirmed significant adsorption of nanoparticles onto the rock surface during the flooding process. This presents a challenge to nano-EOR due to the associated environmental hazards and operation costs.
- According to several studies, the performance of nanofluids is better when applied as a secondary rather than tertiary EOR method. However, the cost of this injection method is a limiting factor.
- Nanofluid stability is a bottle-neck that sometimes can only be improved using dispersions in alcohols or via the use of different stabilizers.
- Nanoparticles favour disjoining pressure to sweep the oil droplets from rock surfaces. According to the studies presented, the main mechanism is wettability alteration. In certain cases, a reduction in water–oil IFT is also found. However, the addition of a surfactant to the nanofluid drastically enhances this reduction, at the same time as improving or worsening the stability of the nanoparticles.
- To avoid excessive adsorption, cationic surfactants are recommended for carbonate and anionic ones for sandstone rocks. However, a general rule cannot be established regarding the best type of nanoparticles according to surfactant type. More work is required in this line of research.
- Polymers, mixed with nanoparticles or used to functionalise them, are usually employed to improve the stability of nanofluids. Nanoparticles help the polymer to increase aqueous viscosity but also reduce apparent oil viscosity and improve its rheological behaviour.
- The synergy of combining nanoparticles, polymers and surfactants leads to promising formulations for EOR. However, designed formulations are complicated and involve high costs. Moreover, the protocol used to prepare the mixtures, especially in the presence of salts, has to be clearly defined.
- The combination of SAILs with nanoparticles is a niche that must be further explored.
- The flooding equipment used in the tests, as well as the type of core and initial conditions, drastically affect oil recoveries with nanofluids, sometimes leading to very optimistic numbers that should be verified.
- A striking absence of information regarding the costs of nano-EOR methods per incremental bbl, a decisive factor for industrial applications, was noted and needs to be addressed.
- Despite the significant number of laboratory studies carried out, the Technology Readiness Level of nano-EOR is still very low; thus, a lot of effort needs to be made to prove the current system in operational environments.
- In summary, there still exists a need for systematic and rigorous works on EOR with nanoparticles, in order to establish general rules for the best design of nanofluids for practical applications.

## Figures and Tables

**Table 1:** Core-flooding tests involving nanoparticles.

Nanoparticles	Concentration (wt%)	T (°C)	Dispersion Media	Oil	Rock Type	Rock Properties	AOR (%OOIP)	Ref
SiO_2_	0.01–0.1	20	3.0 wt% NaCl	North Sea	Berea sandstone (low-permeability)	Ø: 13.93–15.02% µ: 9–35 mD	0–6.14	[18]
SiO_2_	0.01–0.1	20	3.0 wt% NaCl	North Sea	Berea sandstone (high-permeability)	Ø: 20.01–23.20% µ: 156–392 mD	4.26–5.32	[18]
SiO_2_	0.05	25 50 80	3.0 wt% NaCl	Light	Berea sandstone	Ø: 15–19% µ: 100–600 mD	0.81–5.86 ^1^ 3.91–10.87 ^1^ 6.02–10.11 ^1^	[19]
SiO_2_	0.05	r.t.	SSW	North Sea	Berea sandstone	Ø: 15–17 % µ: 100–600 mD	1.98–18.52 ^1^	[20]
SiO_2_	0.01–0.5	r.t.	3.0 wt% NaCl	North Sea	Sandstone	Ø: 19.4–21.7 % µ: 285–587 mD	≤13.3	[21]
SiO_2_	5 × 10^−4^–0.003	50	7500 mg/L NaCl	Shengli oilfield	Feldspar (53%)	Ø: 9.35−11.95% µ: 0.68−0.95 mD	4.48–10.33 ^1^	[22]
SiO_2_	0.02–0.1	r.t.	2–4 wt% NaCl	Paraffin	Sandstone	Ø: 20.72–27.65% µ: 465–603 mD	6.58–11.2	[24]
SiO_2_	0.01–0.1	r.t.	3 wt% NaCl	North Sea	Sandstone	Ø: 20.01–23.20% µ: 156–392 mD	4.26–5.32	[41]
SiO_2_ Fumed/colloidal	0.01–0.1	r.t.	Synthetic North Sea brine ^3^	North Sea	Sandstone	Ø: 17.8–20.8% µ: 285–438 mD	2.36–11.76	[42]
SiO_2_	0.01–3	r.t.	Water ^4^	Mineral oil	Bahariya sandstone	Ø: 26% µ: 378.73 mD	40–79 ^2^	[43]
SiO_2_	0.1	r.t.	Water ^4^	Mineral oil	Sandstone	Ø: 29.87–30.75% µ: 575.82–642.65 mD	65.36–77.94 ^2^	[44]
SiO_2_	0.01–0.1	r.t.	3 wt% NaCl	Heavy	Berea sandstone	Ø: 17.4–20.3% µ: 60 mD	28.56–38.57 ^2^	[45]
SiO_2_ Aerosil 200	0.05–1	100	Water ^4^	Iranian	Carbonate	Ø: 14.379–15.823% µ: 1.0119–1.9552 mD	57.23–65.23 ^2^	[26]
SiO_2_ Aerosil R 816	0–1	100	Water ^4^	Iranian	Carbonate	Ø: 13.268–14.949% µ: 1.0347–1.2446 mD	55.45–80.2 ^2^	[27]
SiO_2_	0.005	26 40 50 60	Water ^4^	Malaysian	Limestone grains	Ø: 41.1–43.2%. µ: 220–240 mD	2.0 2.5 2.8 2.9	[35]
TiO_2_ Anatase	0.01	75	5000 ppm NaCl	Heavy	Sandstone	Ø: 23.7 % µ: 84 mD	80 ^2^	[30]
TiO_2_ Rutile	0–0.05	r.t.	0.1 mol/dm ^3^ NaCl	Mineral oil	Berea sandstone	Ø: 20.82–21.20% µ: 98.73–195.46 mD	30.3–41.8 ^2^	[31]
TiO_2_	0.01–0.1	r.t.	3 wt% NaCl	Heavy	Berea sandstone	Ø: 20.0–24.0% µ: 60 mD	15.69–34.42 ^2^	[45]
TiO_2_	0.005	26 40 50 60	Water^4^	Malaysian	Limestone grains	Ø: 42.3–43.3%. µ: 203–236 mD	3.0 4.1 5.2 6.6	[35]
Aluminosilicate	10^−4^–0.002	60	Formation brine Langgak field	Indonesia	Bentheimer sandstone	Ø: 19.33–20.37% µ: 316.93–633.76mD	4.44–15.59	[32]
Al_2_O_3_	0.01–0.1	r.t.	3 wt% NaCl	Heavy	Berea sandstone	Ø: 19.7–20.4% µ: 60 mD	32.32–38.70 ^2^	[45]
γ-Al_2_O_3_	0.5	r.t.	Water ^4^	Ahwaz oilfield	Carbonate	Ø: 17.3% µ: 0.807 mD	11.25	[33]
Al_2_O_3_	0.005	26 40 50 60	Water ^4^	Malaysian	Limestone grains	Ø: 41.8–43.1%. µ: 205–230 mD	4.5 5.4 7.0 9.9	[35]
SnO_2_	0–0.5	r.t.	SSW	Ahwaz oilfield	Carbonate	Ø: 18% µ: 0.22 mD	39–61 ^1,2^	[34]
NiO	0.01– 0.1	r.t.	3 wt% NaCl	Heavy	Berea sandstone	Ø: 19.0–21.8% µ: 60 mD	31.45–36.20 ^2^	[45]
SiO_2_ + Al_2_O_3_ (50 wt%)	0.01– 0.1	r.t.	3 wt% NaCl	Heavy	Berea sandstone	Ø: 20.2–20.6% µ: 60 mD	24.85–42.29 ^2^	[45]
SiO_2_ + Al_2_O_3_ (50 wt%)	0.05	r.t. 80	16 wt% NaCl	Heavy	Berea sandstone	Ø: 16.1–20.7% µ: 60 mD	29.36 ^2^ 56.72 ^2^	[45]
Magnetite +Magnetic Field	0.8	r.t.	5000 ppm NaCl_3_	Van Gogh/Kuwait	Berea sandstone	Ø: 18.94–21.51%. µ: 60–82 mD	6.32–15.38 ^1^ 8.57–16.20 ^1^	[37]
Fe_3_O_4_ +Magnetic Field	0.05	r.t.	11,000 ppm NaCl	Light	Berea sandstone		15.8 29.4	[38]
SiO_2_ +Smart Water	0.1	85	Formation brine + SO_4_^2−^	Iranian	Carbonate (Dolomite)	Ø: 19.69% µ: 1.0 mD	~11.3	[39]
SiO_2_ +Smart Water	0.05	60	Water + MgCl_2_ + CaCl_2_ + Na_2_SO_4_	Iranian (asphaltenic)	Carbonate		45.6	[40]

^1^ Including water post-flux. ^2^ Secondary flooding with nanofluid. ^3^ Different brines used for core saturation and flooding. ^4^ Brine used for core saturation/secondary flooding.

**Table 2:** Core-flooding tests involving functionalized nanoparticles.

Nanoparticles	Concentration (wt%)	T (°C)	Dispersion Media	Oil	Rock Type	Rock Properties	AOR (%OOIP)	Ref
Nano-hybrid SiO_2_-HAHPAM	0.5 HAHPAM/ 0.5 Silica	85	Synthetic brine with divalent ions	Shengli	Silica sand	Ø: 32.6 % µ: 1498 mD	10.57	[46]
Pyroxenes	0.005	60	2 wt% NaCl	Canada	Berea sandstone	Ø: 19.2 % µ: 63 mD	10.57	[47]
Amphiphilic silica	0.1	90	Water ^1^	Paraffin:Kerosene 10:3	Sandstone	Ø: 20.7–21.4 % µ: 50–800 mD	2.6–10.3	[48]
SiO_2_@Montmorilant @Xanthan	0.1	60	Water ^1^	Gachsaran oilfield	Sandstone	Ø: 15.32 % µ: 30 mD	15.79	[49]
SiO_2_@Montmorilant @Xanthan	0.025	60	Water ^1^	Gachsaran oilfield	Carbonate	Ø: 12.82 % µ: 8.23 mD	11.72	[49]
ZnO/SiO_2_/ Xanthan	0.2	75	Dilute SW	Medium	Carbonate	Ø: 16.85 % µ: 13.15 mD	19.28	[50]
Fe_3_O_4_@Chitosan	0–0.03	r.t.	SW ^2^	Iranian	Carbonate sand		56.7–67.5 ^3^	[51]
Polymer-citrate-coated Fe_3_O_4_	0.02–0.04	85	SSW (mixture of formation water and SW)	Iranian	Silane glass beads	Ø: 23–25% µ: 320–340 mD	12.2–19.7	[52]
Polymer-citrate-coated Fe_3_O_4_	0.04	85	SSW (mixture of formation water and SW)	Iranian	Carbonate	Ø: 17.8% µ: 34.12 mD	28	[52]
KH550-MoS_2_	0.005	55	Synthetic formation brine	Changqing oilfield	Chloritization and illite	Ø: 36.22–38.1% µ: 7.8–8.4 mD	14	[53]
Graphene-based amphiphilic Janus Nanosheets	0.005–0.01	r.t.	4 wt% NaCl + 1 wt% CaCl_2_	China oilfield	Sandstone sand-packs	Ø: 24.8–27.9% µ: 44.5–132 mD	6.7–15.2	[54]
Amine/organosiloxane @Al_2_O_3_/SiO_2_ + Smart Water	0.005	r.t.	SSW +Ca^2+^/SO_4_^2−^	Medium	Carbonate	Ø: 8.57−11.50% µ: 0.54−0.59 mD	3–7.5	[55]

^1^ Brine used for core saturation/secondary flooding. ^2^ Different brines used for core saturation and flooding. ^3^ Secondary flooding with nanofluid.

**Table 3:** Core-flooding tests involving nanoparticles and alcohol.

Nanoparticles	Concentration (g/L)	Dispersion Media	T (°C)	Oil	Rock Type	Rock Properties	AOR (%OOIP)	Ref
SiO_2_ Aerosil R 816	3	Ethanol	r.t.	Light Intermediate	Sandstone	Ø: 18.5% µ: 102 mD	25.43 14.55	[57]
Hydrophobic polysilicon	3	Ethanol	r.t.	Light Intermediate	Sandstone	Ø: 31.29–31.64% µ: 513.59–1476.18 mD	36.67 29.01	[58]
Hydrophobic polysilicon	4	Ethanol	r.t.	Iranian	Sandstone	Ø: 17% µ: 186 mD	32.20	[59]
Neutrally wet polysilicon	3	Ethanol	r.t.	Light Intermediate	Sandstone	Ø: 30.88–31.78% µ: 791.23–939.24 mD	38.75 29.23	[58]
Neutrally wet polysilicon	4	Ethanol	r.t.	Iranian	Sandstone	Ø: 17% µ: 186 mD	28.57	[59]
SiO_2_ -treated by silane	1.5	Propanol	r.t.	Iranian	Sandstone	Ø: 17.34% µ: 108.21 mD	22.5	[60]
Al_2_O_3_	1.5	Propanol	r.t.	Iranian	Sandstone	Ø: 17.45% µ: 110.40 mD	20.2	[60]
Fe_2_O_3_	1.5	Propanol	r.t.	Iranian	Sandstone	Ø: 18.12% µ: 109.32 mD	17.3	[60]

**Table 4:** Core-flooding tests involving nanoparticles and surfactants.

Nanofluid	T (°C)	Dispersion Media	Oil	Rock Type	Rock Properties	AOR (%OOIP)	Ref
0.1 wt% SiO_2_ 2150 ppm SDS	30	Diluted reservoir brine 2000 ppm ^1^	Iranian	Sandstone	Ø: 20% µ: 200 mD	80 ^2^	[61]
0.1 wt% SiO_2_ (Aerosil 200) 2500 ppm SDS	38	Water	Iranian	Sandstone	Ø: 15–16% µ: 40-60 mD	94.7 ^2^	[62]
0.2 wt% SiO_2_ 0.2 wt% SDS	20	Water	Tahe oilfield	Sandstone	Ø: µ: 0.051 μm ^2^	9.11 ^3^	[63]
0.2 wt% SiO_2_ (Aerosil 300) 400 ppm SDS	26	Synthetic brine with divalent ions	Azadeghan	Sandstone	Ø: 18.2 µ: 158.35 mD	14.5	[64]
2000–5000 ppm SiO_2_ (Aerosil 300) 2000 mg/L SDS	r.t.	Water	26 cp (25 °C)	Sandstone sand-packs	Ø: 21.34 µ: 367.96 mD	15.86–17.87 ^3,4^	[65]
2000–10000 ppm SiO_2_ (Aerosil R 816) 2000 mg/L SDS	r.t.	Water	26 cp (25 °C)	Sandstone sand-packs	Ø: 21.34 µ: 367.96 mD	18.26–20.41 ^3,4^	[65]
0.5 wt% Nano-clay 1800 ppm SDS	r.t.	Water ^5^	Iranian	Sandstone	Ø: 18.3–18.5 µ: 281-285 mD	52.3 ^2^	[66]
0.05 wt% ZnO 0.2 wt% SDS	r.t.	Water	Dodecane	Sandstone	Ø: 26.65 µ: 15.74 mD	~15	[67]
100 mg/L Janus graphene oxide (400) 1000 mg/L DTAB	60	Formation brine	Bohai oilfield	Artificial core	Ø: 37.02 µ: 506 mD	19.5 ^3^	[68]
1000 ppm SiO_2_ 5 wt% Cedr extract	25	100,000 ppm NaCl	Reservoir	Sandstone	Ø: 15.4 µ: 1.9 mD	74 ^2^	[69]
97.5 ppm SiO_2_ 110.8 ppm Rhamnolipid	80	1.17 wt% NaCl ^1^	North Azadegan oilfield	Carbonate	Ø: 14.1 µ: 1.45 mD	5.1	[70]
10–500 mg/L SiO_2_ 10–40 mg/L Rhamnolipid	55	3 wt% NaCl	Xinjiang oilfield	Berea sandstone	Ø: 16.45–19.87 µ: 0.0053-3.19 mD	4–25	[71]
0.05 wt% SiO_2_-NH_2_ 0.2 wt% Soloterra 964	65	15 wt% NaCl	Bakken	Berea sandstone	Ø: 18.96 µ: 0.091 μm ^2^	22.37 ^3^	[72]
500 mg/L Amine-terminated SiO_2_ 4000 mg/L Laurel anolamide	30	Formation brine	Xinjiang Oilfield	Three-layer artificial	Ø: 17.6 µ: 100/200/500 mD 50/100/300 mD	26.4–29.2 ^3^	[73]
0.01 wt% SiO_2_ 0.01 wt% Alpha olefin sulfonate	r.t.	18 wt% NaCl	Iranian	Carbonate	Ø: 13.48-15.73 µ: 1.05-1.51 mD	2.5–8.6 ^3^	[75]
0.1 wt% SiO_x_ 0.1 wt% Linear alcohol, C_9-11_, ethoxylate	60	1mM NaCl	Crude oil	Berea Sandstone	Ø: 23.6 µ: 173.2 mD	~55 ^2^	[76]
0.1 wt% SiO_x_ 0.1 wt% Linear alcohol, C_9-11_, ethoxylate	60	1mM NaCl	Crude oil	Edward carbonate	Ø: 24.23 µ: 23.61 mD	~29 ^2,6^	[76]
1000 ppm SiO_2_ 2000 ppm Lauroyl-arginine		Water ^5^	Kupal oilfield	Carbonate	Ø: 13.16 µ: 10.88 mD	13.1	[77]
1000 ppm SiO_2_ 4500 ppm Lauroyl-cysteine		Water ^5^	Kupal oilfield	Carbonate	Ø: 9.38 µ: 8.28 mD	12.7	[77]
1000 ppm SiO_2_ 250 ppm Cocamido propyl betaine	r.t.	19.8 wt% NaCl	Iranian	Carbonate (Dolomite)	Ø: 19.4 µ: 8.4 mD	12.2	[78]
0.1 wt% SiO_2_ 0.03 wt% Linear alkylbenzene sulfonic acid	r.t.	20 wt% NaCl	Iranian	Carbonate	Ø: 19-23 µ: 13-16 mD	~ 4.3–5	[79]
1 wt% Lysine-grafted silica 8000 ppm Jatropha oil derived ^7^	75	2 wt% NaCl	Crude oil	Sand-packs	Ø: 28.55–31.21 µ: 1.16-1.56 mD	33.6 ^3^	[80]
1 wt% Aluminium oxide hydroxide 3mM Sodium dodecylbenzene sulfonate Water:dodecane ratio 1:1 ^7^	60	Water ^5^	Jidong oilfield	Artificial core	Ø: 36–42 µ: ~500 mD	33 ^3^	[81]
0.4 wt% SiO_2_ 0.1 wt% Hexadecyltrimethylammonium bromide Water:biodiesel ratio 9:1 ^7^	50	0.5 wt% NaCl	Shengli oilfield	Man-made	Ø: 18.1-24.6 µ: 100-1100 mD	17.40-50.01	[82]
0.01 wt% SiO_2_ Triton X-100: *n*-dodecul *β*-d-maltoside: *d*-limonene:2-propanol 2:2:2:0.8 ^7^	60	1M CaCl_2_	Gibbs oilfield	Arkose sandstone	Ø: 16.8–17 µ: 26-32 mD	34.3	[84]
0.1 wt% ZnO 0.025 wt% Sodium dodecylbenzene sulfonate + Magnetic Field	95	3 wt% NaCl	Tapis oilfield	Sandstone sand-packs	Ø: 35.40–37.37 µ: 267–284 mD	13.82–15.25 ^3^ 13.98–16.05 ^3^	[86]

^1^ Different brines used for core saturation and flooding. ^2^ Secondary flooding with nanofluid. ^3^ Including water post-flux. ^4^ After surfactant secondary flooding. ^5^ Brine used for core saturation/secondary flooding. ^6^ Lower production than with brine. ^7^ Injected as nanoemulsion.

**Table 5:** Core-flooding tests involving nanoparticles and polymers.

Nanofluid	T (°C)	Dispersion Media	Oil	Rock Type	Rock Properties	AOR (%OOIP)	Ref
0.1 wt% SiO_2_ 0.2 wt% HPAM	90	Water ^1^	Sarawak oilfield	Sandstone	Ø: 15.25–15.31% µ: 165.6–169.1 mD	33 ^2^	[87]
0.05–0.1 wt% SiO_2_ 0.2 wt% HPAM	r.t.	Water ^1^	Iranian	Sandstone	Ø: 18.65–19.30% µ: 10.21–12.90 mD	6.28–7.97	[89]
0.1 wt% Al_2_O_3_ 0.2 wt% HPAM	90	3.41 wt% NaCl	Sarawak oilfield	Sandstone	Ø: 15.25–15.31% µ: 165.6-169.1 mD	37.6 ^2^	[87]
1.9–2.5 wt% TiO_2_ 3150 ppm HPAM	r.t.	Water ^1^	Iranian	Sandstone	Ø: 18.2–18.4% µ: 281–283 mD	40.4–43.3 ^2^	[90]
0.9 wt% Nano-clay 3150 ppm HPAM	r.t.	Water ^1^	Iranian	Sandstone	Ø: 18.7% µ: 284 mD	~42	[91]
1000 ppm MWCNTs 1000 ppm Acrylamide co/terpolymers	85	API Alkaline pH	Mineral oil	Ottawa sand-pack	Ø: 38.28% µ: 95 mD	14.8 10.8	[92]
0.1 wt% Amino-modified SiO_2_/Acrylamide copolymer nanocomposite	85	API Formation brine	Mineral oil	Ottawa sand-pack	Ø: 37.9% µ: 95 mD	17.46 ^2^ 16.20 ^2^	[93]
0.05 wt% Al_2_O_3_ 1 wt% Polyvinylpyrrolidone	r.t.	3 wt% NaCl	North Sea	Berea sandstone	Ø: 14.71% µ: 330 mD	13.34 ^2^	[94]
0.05 wt% TiO_2_ 1 wt% Polyvinylpyrrolidone	r.t.	3 wt% NaCl	North Sea	Berea sandstone	Ø: 14.96 % µ: 118 mD	20.00 ^2^	[94]
0.5–1.5 wt% Al_2_O_3_ 5.0 wt% Potato peel starch	r.t.	Water ^1^	Niger Delta	Niger Delta Berea sandstone	Ø: 22.58–26.70% µ: 291.3–293.1 mD	10.22–12.44 ^3^	[95]
1.33 wt% Al_2_O_3_ 3.0–5.0 wt% Gum Arabic	r.t.	Water ^1^	Niger Delta	Niger Delta Berea sandstone	Ø: 17.89–18.56% µ: 251.7–278.8 mD	5.61-7.81	[95]
0.1–0.5 wt% SiO_2_ 1000–5000 ppm Xanthan gum	30 80	Synthetic formation brine	Heavy	Berea sandstone	Ø: 25.1–26.5% µ: 746–1002 mD	16.29-20.82 ^2^ 18.44 ^2^	[96]
0.2 wt% SiO_2_ 4000 ppm Guar gum	50	Water ^1^	Light	Sandstone	Ø: 16.3% µ: 204 mD	44.3 ^2^	[97]
0.1 wt% Surface-modified montmorillonite 2000 ppm HPAM	r.t.	Water ^1^	Darquain oilfield	Carbonate sand-pack	Ø: 23.3% µ: 294 mD	33 ^4^	[99]
500–1500 ppm of TiO_2_/SiO_2_/poly(acrylamide) nanocomposite + Smart Water	75	Different smart waters	Iranian	Carbonate	Ø: 7.75–9.65% µ: 3.60–4.20 mD	7.4–10.5	[100]

^1^ Brine used for core saturation/secondary flooding. ^2^ Including water post-flux. ^3^ Additional recovery after polymer flooding. ^4^ Secondary flooding with nanofluid.

**Table 6:** Core-flooding tests involving nanoparticles, surfactants and polymers.

Nanofluid	T (°C)	Dispersion Media	Oil	Rock Type	Rock Properties	AOR (%OOIP)	Ref
0.5–2.0 wt% SiO_2_ 0.14 wt% SDS 1000 ppm HPAM	30 90	Water ^1^	Medium	Silica sand-pack	Ø: 29.77 ± 1.34% µ: 1006±86.50 mD	19.25–24.68 ^2^ 21.82 ^2^	[101]
0.2–0.4 wt% PAM-grafted TiO_2_ 0.1 wt% SDS 0.4 wt% HPAM	25	0–3 wt% NaCl	Heavy	Sand-pack	Ø: 34–37% µ: 5276.4–6111.1 mD	~69–86 ^2,3^	[102]
0.5 wt% Colloidal SiO_2_ 0.1 wt% 3-(N,N-dimethylmyristylammonio)propanesulfonate 0.5 wt% PSS-co-MA	25	3 wt% NaCl	Silicone	Sandstone	Ø: 19–22% µ: 138-154 mD	74.2–78.2 ^3^	[103]
0.08 wt% Polymer nanoparticles 0.1 wt%Betaine surfactant	80	Synthetic formation brine	Bakken	Berea sandstone	Ø: 19.22 % µ: 0.08859 μm ^2^	19.95 ^2^	[104]
1.0 wt% SiO_2_ 1.0 wt% PAM 0.22 wt% SDS-Detergent Water:oil ratio 3:1 ^4^	40–90	Water ^1^	Tarapur oilfield	Berea sandstone	Ø: 20.04–22.85 % µ: 498–632 mD	17.49–21 ^2^	[105]
1.0 wt% Clay 1.0 wt% PAM 0.22 wt% SDS-Detergent Water:oil ratio 3:1 ^4^	40–90	Water ^1^	Tarapur oilfield	Berea sandstone	Ø: 20.17–23.05 % µ: 584–703 mD	17.25–20.12 ^2^	[105]
0.25 wt% SiO_2_ 1.5 wt% Carboxy methyl cellulose 825 ppm SDBS Water:oil ratio 9:1 ^4^	r.t.	Water ^1^	Light mineral	Sand pack	Ø: 31.48% µ: 1247 mD	24.81 ^2^	[106]
0.025 wt% SiO_2_ 0.05 wt% HPAM 0.1 wt% N,N′-bis((dimethyltetradecyl)-1,6-hexanediammonium bromide) Water:heptane ratio 9:1 ^4^	r.t.	Water ^1^	Ahmedabad oilfield	Sandstone	Ø: 17–18% µ: 350-400 mD	26.25 ^2^	[107]
0.05 wt% Al_2_O_3_ 0.5 wt% 1-Dodecylpyridinium chloride 1 wt% Polyvinylpyrrolidone	r.t.	0.5 wt% NaCl	Light	Carbonate	Ø: 18.08% µ: 22.02 mD	11.96	[110]
0.05 wt% Al_2_O_3_ 0.05 wt% 1-dodecyl-3-methylimidazolium chloride 1 wt% Polyvinylpyrrolidone	r.t.	5 wt% NaCl	Light	Carbonate	Ø: 14.57% µ: 13.97 mD	14.8	[111]

^1^ Brine used for core saturation/secondary flooding. ^2^ Including water post-flux. ^3^ Secondary flooding with nanofluid. ^4^ Injected as nanoemulsion.

## Nomenclature

AOR	Additional oil recovery
AOS	Alpha olefin sulfonate
API	American Petroleum Institute
CAPD	Cocamido propyl betaine
CMC	Carboxy methyl cellulose
CNT	Carbon nanotubes
CTAB	Hexadecyltrimethylammonium bromide
DTAB	Dodecyltrimethylammonium bromide
EOR	Enhanced oil recovery
GA	Gum Arabic
HAHPAM	Hydrophobic association of partially hydrolysed polyacryamide
HPAM	Hydrolysed polyacrylamide
IFT	Interfacial tension
JGO	Janus graphene oxide
MWCNTs	Multi-wall carbon nanotubes
OOIP	Original oil in place
PAM	Polyacrylamide
PSP	Potato peel starch
PVP	Polyvinylpyrrolidone
r.t.	Room temperature
SAILs	Surface-active ionic liquids
SEM	Scanning electron microscopy
SDBS	Sodium dodecylbenzene sulfonate
SDS	Sodium dodecyl sulphate
SSW	Synthetic sea water
SW	Sea water
